# Comparison of the antioxidant capacity of sesamol esters in gelled emulsion and non-gelled emulsion

**DOI:** 10.1016/j.fochx.2023.100700

**Published:** 2023-05-03

**Authors:** Malihe Keramat, Mohammad-Taghi Golmakani, Mehrdad Niakousari, Mohamad Reza Toorani

**Affiliations:** Department of Food Science and Technology, School of Agriculture, Shiraz University, Shiraz, Iran

**Keywords:** Gelled emulsion, Mass transfer, Oxidative stability, Sesamol esters

## Abstract

The antioxidant capacity of sesamol esters in gelled emulsion was investigated in comparison with non-gelled emulsion to assess the role of mass transfer on their antioxidant capacity. Initiation phase and propagation phase kinetic parameters of peroxidation was calculated using a sigmoidal model. Sesamol esters showed higher antioxidant activity than sesamol in gelled emulsion and non-gelled emulsion. Sesamyl acetate, sesamyl butyrate, and sesamyl hexanoate had no synergistic effect with sesamol in gelled emulsion, while in non-gelled emulsion sesamyl butyrate exhibited a slight synergistic effect with sesamol. The antioxidant activity of sesamyl acetate and sesamyl hexanoate in non-gelled emulsion samples were higher than those of gelled emulsion samples, while sesamyl butyrate exhibited higher antioxidant activity in gelled emulsion than that of non-gelled emulsion. The cut-off effect hypothesis was observed in gelled emulsion, while this hypothesis was disappeared in non-gelled emulsion. During propagation phase, sesamol esters remained active and exhibited inhibitory effect.

## Highlights

• Efficiency of sesamol esters was evaluated in gelled emulsion and non-gelled emulsion.
• Sesamol esters exhibited higher efficiency than sesamol on improving oxidative stability.
• Cut-off effect was observed in gelled emulsion, while it was vanished in non-gelled emulsion.
• The efficiency of sesamyl acetate and sesamyl hexanoate reduced in gelled emulsion.
• Sesamyl butyrate showed higher efficiency in gelled emulsion than non-gelled emulsion.

## Graphical abstract

**Figure:**
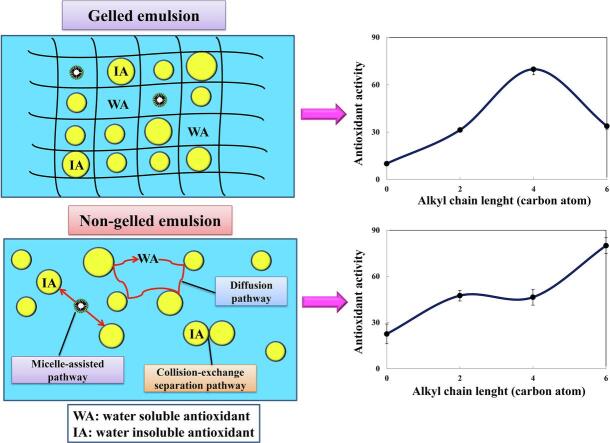

## 1 Introduction

Lipid oxidation is an important reaction that occurs in oil-in-water (O/W) emulsions. Consisting of small oil droplets dispersed in a water phase, O/W emulsions expose a significantly larger surface area to the surrounding water phase, as compared to the bulk oil. Since pro-oxidant compounds in the aqueous phase come into contact with unsaturated fatty acids at the interfacial area, it has been proposed that the peroxidation process initiates and propagates at the interfacial area. Therefore, antioxidants that can accumulate at the interfacial area can efficiently reduce lipid oxidation (McClements & Decker, 2018).

Sesamol is a phenolic compound present in sesame seed oil (Hwang, Winkler‐Moser, Bakota, Berhow, & Liu, 2013). This compound can significantly scavenge lipid peroxyl, hydroxyl, organo-haloperoxyl, one-electron oxidizing, and tryptophanyl radicals (Joshi, Kumar, Satyamoorthy, Unnikrisnan, & Mukherjee, 2005). Recent studies have suggested that the esterification of carboxyl group of phenolic compounds with fatty alcohols or esterification of their hydroxyl groups with fatty acids can alter the hydrophilic - lipophilic balance (HLB) of phenolic compounds and enhance their affinity for locating at the interfacial area of emulsions (Alemán et al., 2015, Laguna et al., 2020). Alkyl chain length (Laguerre et al., 2010), alkyl chain unsaturation degree (Xu et al., 2022), dispersed phase volume ratio (Almeida et al., 2016), physicochemical characteristics of emulsion system (Alemán et al., 2015), pH (da Silveira et al., 2021), droplet size (Almeida et al., 2016), surfactant type (Sørensen, Villeneuve, & Jacobsen, 2017), and presence of surfactant micelles (Panya et al., 2012) can affect the efficiency of phenolic compounds esters in O/W emulsions.

Another important parameter that can impact the effectiveness of antioxidants in reducing emulsion oxidation is their ability to move to the interfacial area, where peroxidation occurs. This parameter is often ignored in determining the efficiency of phenolic compounds esters in emulsions. Laguerre, Bily, Roller, and Birtić (2017) suggested that in O/W emulsions, hydrophilic and lipophilic antioxidants transfer between oil droplets through different pathways. Hydrophilic antioxidants are hypothesized to only be transferred within emulsion by diffusing through the water phase. Hydrophobic antioxidants are assumed to be transferred via micelle-assisted transfer pathway or via the collision of adjacent oil droplets. The transfer rate would be higher by the micelle-assisted transfer pathway and depend on the size and concentration of the micelles.

Gelled emulsion is a specific class of emulsions in which the emulsion is gellified via a gelling agent (Lee et al., 2016). Gelled emulsions can mimic water holding capacity and hardness of solid fats in meat products (Poyato, Ansorena, Berasategi, Navarro-Blasco, & Astiasarán, 2014). Compared to conventional emulsions, gelled emulsions can modulate and prolong gastric and/or intestinal drug release due to the increased protective effects against gastric and intestinal phases via the gel network (Guo, Bellissimo, & Rousseau, 2017). Gelled emulsion is more viscous than non-gelled emulsion. Viscosity can affect the transfer rate of molecular species in O/W emulsion. The high viscosity of gelled emulsion can limit the transfer of pro-oxidant compounds present in the water phase to the interfacial area (Sato, Moraes, & Cunha, 2014).

As far as we know, esterification of sesamol for improving its interfacial activity in dispersed systems has not yet been investigated. In addition, the efficiency of esterified antioxidants in gelled emulsions has not been investigated. The objective of this study was to compare the inhibitory effect of sesamol and sesamol esters as well as combination of sesamol + sesamol esters with different hydrophilic-lipophilic balance values in gelled emulsion in comparison with the non-gelled emulsion. A sigmoidal model was also applied for determining initiation phase and propagation phase kinetic parameters of peroxidation process.

## 2 Materials and methods

### 2.1 Materials

Sunflower oil was supplied from a local market. Butyric anhydride (>98%), acetic anhydride (>98%), ferrous sulfate, 2,2-diphenyl-1-picrylhydrazyl (DPPH) radical, barium chloride, sesamol (>98%), and ammonium thiocyanate were supplied from Sigma-Aldrich Company (St. Louis, MO). Methanol, hydrochloric acid, chloroform, and *n*-heptane were supplied from Merck Company (Darmstadt, Germany). Hexanoic anhydride (>97%) was supplied from Acros Company (New Jersey, USA).

### 2.2 Production of sesamol esters

To produce sesamyl acetate (SA), sesamyl butyrate (SB), and sesamyl hexanoate (SH), sesamol was blended separately with different anhydrides at 1:1.5 M ratio (sesamol:anhydride). The mixtures were stirred at 70 °C until completion of the reaction. Then, excess of anhydrides and produced acids were evaporated from the mixture by evaporation under vacuum. Sesamol esters purity (>95%) was measured via gas chromatography/flame ionization detector by the method of Keramat, Golmakani, and Toorani (2021).

### 2.3 Identification of sesamol esters

Fourier-transform infrared spectroscopy (FTIR) was carried out with a Perkin–Elmer FTIR instrument (Tensor II, Bruker, Germany). The spectral range was 4000 to 400 cm^−1^. Gas chromatography/mass spectrometry (GC/MS) was performed with an Agilent instrument (GC model 7890A; MS model 5977B; Agilent Technologies, Santa Clara, CA) with a DB-225 ms capillary column (30 m; 0.25 mm internal diameter; 0.25 μm film thickness) (Keramat, Golmakani, Durand, Villeneuve, & Hosseini, 2021). Carbon nuclear magnetic resonance (^13^C NMR), and hydrogen nuclear magnetic resonance (^1^H NMR) spectral data were determined on a 400 MHz (Bruker Avance-Ш, Karlsruhe, Germany) spectrometer at 25°.

### 2.4 Partition coefficient (log *P* value)

The log *P* value of sesamol and its esters were measured via the Molinspiration software (Molinspiration Cheminformatics, Bratislava, Slovak Republic).

### 2.5 Radical scavenging capacity

DPPH assay was applied for determining radical scavenging capacity of sesamol and its esters. In brief, 200 µL methanolic solution of sesamol or sesamol esters at various concentrations (6090, 609, 60.9, 6.09, and 0.609 µM) were blended with 1800 µL methanolic DPPH solution (0.2 mM). Then, samples were kept in the darkness for 60 min. After that, the absorbance values were determined at 517 nm against blank (methanol) using a spectrophotometer (UV*-*9200*/*VIS*-*7220G, Beifen-Ruili, Beijing, China). Radical scavenging capacity of sesamol and sesamol esters was stated in terms of IC_50_ value. This parameter is described as the minimum concentration of sesamol or sesamol esters that is needed for 50% inhibition of DPPH radical (Mimica-Dukić, Božin, Soković, Mihajlović, & Matavulj, 2003).

### 2.6 Oil purification

Sunflower oil purification was done via an adsorption chromatography column method using the method of Farhoosh and Nyström (2018). Briefly, aluminum oxide 60 (60 g) was heated at 200 °C for 180 min. Then, the activated aluminum oxide 60 was poured into a glass column (36 cm × 2.9 cm I.D.). After cooling aluminum oxide 60 to room temperature, the sunflower oil (90 g) was passed through the column using a vacuum pump.

### 2.7 Preparation of O/W emulsion

Emulsions were produced by emulsion phase inversion method. The oil:water ratio was 1:10. The Tween 80 concentration in O/W emulsion was 4.35% (w/v). At first, sesamol, sesamol esters, and combination of sesamol + sesamol esters (50/50%, w/w) were dissolved in acetone and separately incorporated into sunflower oil at 0.046 mmol L^-1^ oil. The solvent was evaporated from samples by a nitrogen stream. Then, Tween 80 was mixed with sunflower oil and stirred for 30 min at 750 rpm. After that, potassium phosphate buffer solution (0.04 mol L^-1^; pH 7) was added to the oil with 0.3 mL min^−1^ flow rate, while stirring the mixture at 750 rpm (Ostertag, Weiss, & McClements, 2012). A dynamic light scattering instrument (SZ-100 nanopartica series, Horiba Ltd., Kyoto, Japan) instrument was used for determining the droplet size of the emulsion. Before analysis, the emulsion was diluted 100-times by potassium phosphate buffer. The average droplet size of the emulsion was 300 ± 5.60 nm.

### 2.8 Production of gelled emulsion

Emulsions produced by phase inversion method (according to the method stated in section 2.7) were used for production of gelled emulsion. For emulsion samples used for preparation of gelled emulsion, potassium chloride was incorporated into the water phase at 1.25% (w/w). The method described by Kamlow, Spyropoulos, and Mills (2021) was used for production of gelled emulsion. In brief, emulsions were heated for 5 min at 80 °C. Then, kappa-carrageenan (2%, w/w) was added to the emulsions as gelling agent. After that, the emulsions were heated for 10 min at 80 °C. Finally, gelled emulsions were cooled down to room temperature and stored at refrigerator for 24 h.

### 2.9 Monitoring oxidation

Gelled emulsion and non-gelled emulsions (20 g) were kept in an incubator at 60 °C. The production of hydroperoxides was determined by measuring peroxide value (PV) during storage period. Hydroperoxide extraction from gelled emulsion and non-gelled emulsion samples was performed using the method of Sato et al. (2014). In brief, gelled emulsion or non-gelled emulsion sample (0.3 g) was mixed with a mixture of isooctane:2-propanol (1.5 mL, 3:2, v/v). Then, sample was vortexed for 30 s, and then centrifuged at 25 °C (2000 × g for 120 s). The upper phase of centrifuged sample was collected for measuring PV. The PV was measured using the method of Shantha and Decker (1994) by measuring absorbance at 500 nm. In brief, 0.2 mL of the upper phase of centrifuged sample was blended with a mixture of methanol:chloroform (2.8 mL, 3:7, v/v). Then, ammonium thiocyanate solution (15 µL, 30%, w/v) and Iron (II) chloride solution (15 µL) were added to each sample. To produce iron (II) chloride solution, iron (II) sulfate heptahydrate solution (1 mL, 1%, w/v) was mixed with barium chloride solution (1 mL, 0.8%, w/v) and HCl (40 µL, 10 N). After adding ammonium thiocyanate and iron (II) chloride solutions, the sample was kept at room temperature for 5 min. Then, the absorbance value was measured at 510 nm using a spectrophotometer (UV-9200/VIS-7220G, Beifen-Ruili, Beijing, China). Lipid hydroperoxide concentration (mM) was determined by cumene hydroperoxide standard curve.

### 2.10 Kinetic study

Changes in PVs (mM) of gelled emulsion and non-gelled emulsion samples were plotted versus time (t, h) to obtain kinetic curves of hydroperoxide *(ROOH)* formation. During the initiation phase, kinetic curves of *ROOH* production fitted well with the linear equation (Eq. (1)) (Farhoosh, 2018).

(1) $$\left[ROOH\right]=W_{\mathrm{IP}}\left(t\right)+{\left[\mathrm{ROOH}\right]}_{0}$$

where [*ROOH*]_0_ (mM) is *ROOH* concentration at the beginning of the experiment and *W_IP_* (mM h^−1^) is the pseudo-zero order rate constant during the initiation phase (Farhoosh, 2018).

The kinetic curves of *ROOH* production during the initiation and propagation phases were described by the sigmoidal equation ((Eq. (2)).

(2) $$\left[ROOH\right]=\frac{W_{f}}{\exp\left[W_{f}\left(C-t\right)\right]+W_{d}}$$

where *W_f_* (h^−1^) is a pseudo-first order rate constant of *ROOH* production, *W_d_* (mM^−1^h^−1^) is a pseudo-second order rate constant of *ROOH* decomposition in the propagation phase, and *C* (mM^−1^) is an integration constant (Farhoosh, 2018).

The *[ROOH]*_max_ (mM) parameter was calculated using Eq. (3).

(3) $${\left[\mathrm{ROOH}\right]}_{\max}={\text{lim}}_{t\to \infty }\left\{\frac{W_{f}}{\exp\left[W_{f}\left(C-t\right)\right]+W_{d}}\right\}=\frac{W_{f}}{W_{d}}$$

The turning point (*T_p_*, h) was calculated using Eq. (4). In *T_p_*, the *ROOH* production rate reaches its maximum value in the propagation phase.

(4) $$T_{p}\;=\;\frac{W_{f}C\;-\;\ln W_{d}}{W_{f}}$$

Highest rate of *ROOH* formation during the propagation phase (*K*_max_, mM h^−1^) was measured by Eq. (5).

(5) $$K_{\max}={\left(\frac{d[ROOH]}{\mathrm{dt}}\right)}_{\max}=\frac{W_{f}^{2}}{4W_{d}}$$

The normalized form of *K_max_* (*K_n_*, h^−1^) was measured by Eq. (6).

(6) $$K_{n}=\frac{K_{\max}}{{\left[\mathrm{ROOH}\right]}_{\max}}$$

*IP* (h) was calculated using Eq. (7).

(7) $$\mathrm{IP}=\frac{W_{f}\left(2-W_{f}C+\ln W_{d}\right)-4{\left[\mathrm{ROOH}\right]}_{0}W_{d}}{4W_{\mathrm{IP}}W_{d}-W_{c}^{2}}$$

The end time of the propagation phase (*ET_pp_*, h) was measured by Eq. (8).

(8) $$ET_{\mathrm{pp}}=\frac{4W_{d}R_{\max}-W_{f}K_{n}\left(2-W_{f}C+\ln W_{d}\right)}{4W_{d}K_{\max}K_{n}}$$

Propagation period (*PP*, h) was measured by Eq. (9).

(9) $$\mathrm{PP}=ET_{\mathrm{pp}}--IP$$

The resistance to the oxidation in the initiation phase (*P_r_*, h^2^ mM^−1^) was measured by Eq. (10).

(10) $$P_{r}=\frac{\mathrm{IP}}{k_{i}}$$

The stabilization factor (*E_i_*) which indicates the effectiveness of an antioxidant to scavenge peroxyl radicals in the initiation phase was measured by Eq. (11).

(11) $$E_{i}=\frac{IP_{\mathrm{AH}}}{IP_{C}}$$

where *IP_AH_* is the *IP* of samples containing antioxidant and *IP_C_* is the *IP* of the control sample.

Oxidation rate ratio in the initiation phase (*ORR_i_*) was measured by Eq. (12).

(12) $$\mathrm{OR}R_{i}=\frac{W_{\mathrm{IP},\;AH}}{W_{\mathrm{IP},\;C}}$$

where *W_IP, AH_* is the *W_IP_* value of samples containing antioxidant and *W_IP, C_* is the *W_IP_* value of the control sample.

Antioxidant activity (A) was determined by Eq. (13).

(13) $$A=\frac{E_{i}}{\mathrm{OR}R_{i}}=\frac{P_{r,AH}}{P_{r,C}}$$

Efficiency of antioxidants in the propagation phase (*E_p_*) was measured by Eq. (14).

(14) $$E_{p}\;=\;\frac{PP_{\mathrm{AH}}}{PP_{C\;}}$$

where *PP_AH_* is the *PP* value of samples containing antioxidant and *PP_c_* is the *PP* value of the control sample.

The oxidation rate ratio of *ROOH* production (*ORR_f_*) was measured by Eq. (15).

(15) $$\mathrm{OR}R_{f}\;=\;\frac{W_{f,\;A}}{W_{f,\;B}}$$

where *W_f, AH_* is the *W_f_* value of samples containing antioxidant and *W_f, C_* is the *W_f_* value of the control sample.

The oxidation rate ratio of *ROOHs* decomposition (*ORR_d_*) in the propagation phase was measured by Eq. (16).

(16) $$\mathrm{OR}R_{d}\;=\;\frac{W_{d,\;AH}}{W_{d,\;C}}$$

where *W_d, AH_* is *W_d_* value of samples containing antioxidant and *W_d, B_* is the *W_d_* value of the control samples

Inhibitory effect of antioxidant molecule against the *ROOHs* formation (*IE_f_*) was measured by Eq. (17).

(17) $$IE_{f}\;=\;\frac{E_{p}}{\mathrm{OR}R_{f}}$$

The inhibitory effect of antioxidant molecule against the *ROOHs* decomposition (*IE_d_*) was measured by Eq. (18) (Farhoosh, 2021).

(18) $$IE_{d}\;=\;\frac{E_{p}}{\mathrm{OR}R_{d}}$$

### 2.11 Statistical analysis

All assays were performed in triplicate. Comparing the mean values were done by Duncan’s multiple range test (*P* < 0.05). Significant differences among the mean values were assessed via one-way analysis of variance. To compare emulsion gel with non-gelled emulsions, two-way ANOVA was used. Also, two-way ANOVA was used to show how addition of different antioxidants and entrapping O/W emulsion in a gel network, in combination, can alter kinetic parameters. SPSS 16 software (SPSS Inc., Chicago, IL) was used for statistical analysis. CurveExpert and Microsoft Office Excel software were used for regression analyses.

## 3 Results and discussion

### 3.1 Confirming the production of sesamol esters

**Fig. 1:**
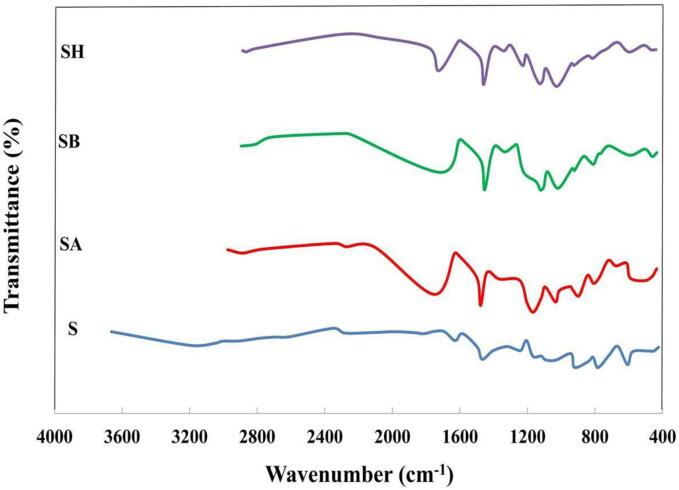
FTIR spectra of sesamol esters. (S: sesamol, SA: sesamyl acetate, SB: sesamyl butyrate, and SH: sesamyl hexanoate).

FTIR spectra of sesamol and sesamol esters are shown in Fig. 1. A broad band around 3004–3663 cm^−1^, which is related to the O—H stretching vibration of the sesamol hydroxyl group was disappeared in the spectra of sesamol esters. The band in the region of 1613–1614 cm^−1^ in the spectra of sesamol esters is related to the carbonyl group (C=O). The band at 1094–1140 cm^−1^ is due to the C—O—C symmetric stretching vibration of the ester linkage. Mass spectra of sesamol esters are shown in Fig. S1. SA, SB, and SH molecular ions were placed at 180.0, 208.1, and 236.1 *m*/*z*, respectively.

^13^C NMR data of sesamol esters are shown in Table 1. Downward chemical shifts were found for 3-position carbon (Δ*δ* of 2.55, 2.58, and 2.59 for SA, SB, and SH, respectively). Upward chemical shifts (*δ*) were found for 2-position carbon (Δ*δ* of 7.23, 7.24, and 7.20, for SA, SB, and SH, respectively) and 4-position carbon (Δ*δ* of 3.43, 3.38, and 3.37 for SA, SB, and SH, respectively). 2-Position carbon and 4-position carbon are the adjacent carbons of the ester bond. The chemical shifts recorded at 169.77–172.60 ppm in sesamol esters are related to the ester group.

**Table 1:** Carbon nuclear magnetic resonance (^13^C NMR) data of sesamol esters.

**3,4-methylenedioxyphenyl unit**	S*	**SA**	**SB**	**SH**
1	108.20^**^	107.93	107.96	107.91
2	106.68	113.91	113.92	113.88
3	150.55	148.00	147.97	147.96
4	98.31	101.74	101.69	101.68
5	148.27	145.35	145.27	145.25
6	141.58	144.98	145.02	145.04
7	101.19	103.73	103.78	103.75

**Acyl chain**				
1ʹ	—	169.77	172.49	172.60
2ʹ	—	20.91	36.11	34.22
3ʹ	—	—	18.47	24.62
4ʹ	—	—	13.66	31.26
5ʹ	—	—	—	22.47
6ʹ	—	—	—	13.93

*S: sesamol, SA: sesamyl acetate, SB: sesamyl butyrate, and SH: sesamyl hexanoate.

^**^Chemical shift (*δ*, ppm).

^1^H NMR data of sesamol esters are shown in Table 2. The chemical shifts (*δ*) between *δ* 0.95 and 2.70 were ascribed to alkyl chain of fatty acids. Also, chemical shifts between *δ* 5.94 and 6.78 were attributed to 3,4-methylenedioxyphenyl unit in sesamol. Differences were observed for the chemical shifts of the adjacent hydrogens of the ester group (Δ*δ* of 0.09, 0.07, and 0.35 ppm for 1-, 2-, and 4-position protons, respectively), compared to sesamol.

**Table 2:** Hydrogen nuclear magnetic resonance (^1^H NMR) data of sesamol esters.

**3,4-methylenedioxyphenyl unit**	S*	**SA**	**SB**	**SH**
1	6.69 (dd, *J* = 8.6, 0.5 Hz, 1H)^**^	6.78 (dd, *J* = 8.7, 0.5 Hz, 1H)	6.78 (dd, *J* = 8.7, 0.5 Hz, 1H)	6.78 (dd, *J* = 8.7, 0.5 Hz, 1H)
2	6.46 (dd, *J* = 8.6, 2.7 Hz, 1H)	6.53 (dd, *J* = 8.7, 2.6 Hz, 1H)	6.53 (dd, *J* = 8.7, 2.6 Hz, 1H)	6.53 (dd, *J* = 8.7, 2.6 Hz, 1H)
4	6.27 (dd, *J* = 2.7, 0.5 Hz, 1H)	6.62 (dd, *J* =, 2.6, 0.5 Hz, 1H)	6.62 (dd, *J* =, 2.6, 0.5 Hz, 1H)	6.62 (dd, *J* =, 2.6, 0.5 Hz, 1H)
7	5.94 (d, *J* = 10.1 Hz, 2H)	5.99 (d, *J* = 10.2 Hz, 2H)	5.99 (d, *J* = 10.2 Hz, 2H)	6.00 (d, *J* = 10 Hz, 2H)

**Acyl chain**				
2ʹ	—	1.05 (t, *J* = 7.3 Hz, 3H)	2.53 (t, *J* = 7.4 Hz, 2H)	2.70 (t, *J* = 7.4 Hz, 2H)
3ʹ	—	—	1.80 (tq, *J* = 7.4, 7.3 Hz, 2H)	2.54 (tt, *J* = 7.7, 7.4 Hz, 2H)
4ʹ	—	—	1.05 (t, *J* = 7.3 Hz, 3H)	1.38 (tt, *J* = 7.7, 7.0 Hz, 2H)
5ʹ	—	—	—	1.74 (h, *J* = 7.0 Hz, 2H)
6ʹ	—	—	—	0.95 (t, *J* = 7.0 Hz, 3H)

^**^Chemical shift (*δ*, ppm).

*S: sesamol, SA: sesamyl acetate, SB: sesamyl butyrate, and SH: sesamyl hexanoate).

### 3.2 Radical scavenging capacity of sesamol esters

The results of DPPH assay showed that sesamol, SA, SB, and SH, showed radical scavenging capacity with IC_50_ values of 0.42 ± 0.01, 7.61 ± 0.95, 5.04 ± 0.42, and 55.27 ± 7.41 mmol L^-1^, respectively. Accordingly, sesamol showed better radical scavenging capacity than its esters. This can be related to the higher hydrogen-donating capacity of hydroxyl group than the substituted carboxylate ester group. Similarly, quercetin (Oh, Ambigaipalan, & Shahidi, 2019), resveratrol (Oh & Shahidi, 2017), and epigallocatechin (Ambigaipalan, Oh, & Shahidi, 2020) showed higher radical scavenging capacity than their esterified homologous. SH exhibited lower radical scavenging capacity than SA and SB. This can be attributed to the lower accessibility of SH active groups to the radical site in DPPH molecule due to the higher steric hindrance of SH. In DPPH assay, steric accessibility is an important parameter since a molecular rotation is needed for reactive groups in sesamol esters molecules to orient towards the radical site in DPPH radical (Xie & Schaich, 2014).

### 3.3 Oxidation kinetic parameters in the initiation phase

In the control sample (sample without antioxidant), the *IP* (induction period) value of gelled emulsion sample was 2.93-fold higher than the non-gelled emulsion sample (Table 3). Also, the *W_IP_* (pseudo-zero order rate constant) value of non-gelled emulsion sample was 2.94-fold higher than the gelled emulsion sample. The *P_r_* value of the control gelled emulsion sample was 8.67-fold higher than the corresponding non-gelled emulsion sample, which indicates the better resistance of gelled emulsion sample to peroxidation during the initiation phase. The lower oxidation rate of gelled emulsion can be attributed to the higher viscosity of the water phase, which can limit the diffusion of pro-oxidant molecules to the interfacial area (Sun, Gunasekaran, & Richards, 2007). According to Table 3, gelled emulsion samples containing sesamol, sesamol esters, and combination of sesamol + sesamol esters showed higher *IP* values in comparison with the corresponding non-gelled emulsion samples. The *E_i_* value can apply as a symbol for indicating the hydrogen donating mechanism of antioxidant molecule (Toorani & Golmakani, 2022). Higher *E_i_* values shows the higher capacity of antioxidant molecules in taking part in the inhibitory reaction (AH + ROO^•^ → ROOH + A^•^). Sesamol esters showed higher *E_i_* values than sesamol in gelled emulsion and non-gelled emulsion samples. The *ORR_i_* value is an emblem of the electron transfer mechanism of the antioxidant compounds. The lower *ORR_i_* value shows higher capacity of antioxidant radical to take part in the inhibitory sub-reaction (A^•^ + ROO^•^ → A-OOR). SA, SB, and SH, showed lower *ORR_i_* value than sesamol in gelled emulsion and non-gelled emulsion samples. This indicates higher participation of sesamol esters in quenching ROO^•^. The *AA* value unifies *E_i_* value and *ORR_i_* value and simultaneously investigates electron transfer and hydrogen donating capacities of antioxidant compounds. According to Table 1, esterification of sesamol with different anhydrides improved the *AA* value of sesamol in gelled emulsion and non-gelled emulsion. The initiation phase of peroxidation process mostly occurs in the interfacial area of O/W emulsion. Polar antioxidants have lower affinity than their non-polar counterparts for accumulating at the interfacial area. The log *P* values of sesamol, SA, SB, and SH, were 1.35, 1.38, 2.61, and 3.62, respectively. Therefore, sesamol esters are less polar than sesamol. Thus, the higher *AA* value of sesamol esters in gelled emulsion and non-gelled emulsion can be attributed to the higher affinity of sesamol esters for localizing at oil–water interface, which can enhance their efficiency to inhibit peroxidation process.

**Table 3:** Oxidation kinetic parameters of the initiation stage of gelled and non-gelled emulsion samples containing sesamol esters.

**Kinetic parameter**	**Control**	S*	**SA**	**SB**	**SH**	**S + SA**	**S + SB**	**S + SH**
**Gelled emulsion**								
IP (h)^**^	33.98 ± 2.49^f***^	163.73 ± 3.69^d^	211.46 ± 4.34^c^	382.44 ± 15.83^a^	301.22 ± 5.92^b^	167.77 ± 1.64^d^	194.27 ± 0.14^c^	110.15 ± 3.25^e^
***W_IP_ ×* 10^2^**	11.89 ± 0.05^f^	5.71 ± 0.07^d^	2.40 ± 0.09^c^	2.21 ± 0.03^a^	3.14 ± 0.48^b^	5.78 ± 0.41^d^	4.04 ± 0.04 ^cd^	11.23 ± 0.08^e^
***E_i_***	–	4.84 ± 0.46 ^cd^	6.29 ± 1.74^c^	11.30 ± 1.29^a^	8.90 ± 0.83^b^	4.97 ± 0.86 ^cd^	5.73 ± 0.42^c^	3.25 ± 0.14^d^
ORR_i_ * 10	–	4.80 ± 0.04^b^	2.01 ± 0.09^d^	1.87 ± 0.87^d^	2.64 ± 0.39 ^cd^	4.86 ± 0.32^b^	3.40 ± 0.04^c^	9.45 ± 0.10^a^
***AA***	–	10.06 ± 0.88^b^	31.41 ± 0.97^b^	69.81 ± 3.51^a^	33.82 ± 1.91^ab^	10.18 ± 1.09^b^	16.85 ± 1.44^b^	3.44 ± 0.19^b^
***P_r_ ×* 10^-2^**	2.86 ± 0.22^d^	28.67 ± 0.29 ^cd^	88.71 ± 12.55^b^	195.23 ± 17.51^a^	96.92 ± 12.95^b^	29.00 ± 0.86 ^cd^	48.03 ± 0.39^c^	9.80 ± 0.22^d^

**Non-gelled emulsion**								
***IP* (h)**	11.60 ± 2.41^b*^	88.35 ± 4.89^a^	115.46 ± 4.34^a^	111.39 ± 8.25^a^	122.98 ± 8.25^a^	24.83 ± 4.79^b^	106.82 ± 1.42^a^	98.76 ± 4.36^a^
***W_IP_ ×* 10^2^**	34.91 ± 1.41^a^	12.14 ± 0.21^a^	7.55 ± 0.02^d^	7.38 ± 1.09^de^	5.37 ± 1.94^de^	23.97 ± 2.09^b^	5.97 ± 0.19^de^	4.50 ± 0.01^a^
***E_i_***	–	7.83 ± 0.25^a^	10.22 ± 2.50^a^	9.89 ± 2.77^a^	10.88 ± 2.63^a^	2.23 ± 0.88^b^	9.40 ± 1.84^a^	8.28 ± 2.27^a^
*ORR_i_* *10	–	3.48 ± 0.08^b^	2.16 ± 0.09^c^	2.11 ± 0.23^c^	1.55 ± 0.62^c^	6.88 ± 0.88^a^	1.71 ± 0.17^c^	1.29 ± 0.05^c^
***AA***	–	22.59 ± 6.43^bc^	47.46 ± 3.47^abc^	46.45 ± 5.14^abc^	79.99 ± 5.20^a^	3.35 ± 1.70^c^	55.05 ± 6.06^abc^	63.95 ± 3.54^ab^
***P_r_* × 10^-2^**	0.33 ± 0.08^c^	7.28 ± 0.28^bc^	15.29 ± 0.57^ab^	15.18 ± 1.12^ab^	24.69 ± 2.71^a^	1.05 ± 0.29^c^	17.92 ± 0.81^ab^	21.98 ± 1.76^a^

^***^Mean ± SD (*n* = 3). In each row, means with different superscript letters are significantly different (*P* < 0.05).

*S: sesamol, SA: sesamyl acetate, SB: sesamyl butyrate, and SH: sesamyl hexanoate.

^**^IP: induction period, *W_IP_*: pseudo-zero order rate constant at the initiation stage, *E_i_*: antioxidant effectiveness during the initiation stage, *ORR_i_*: oxidation rate ratio during the initiation stage, *AA*: antioxidant activity, and *P_r_*: oxidation resistance.

The *P_r_* value simultaneously investigates *IP* and *W_IP_*. The higher value of this parameter shows the higher resistance of samples to the production of ROOH during the initiation phase. According to the two-way ANOVA results, addition of sesamol and sesamol esters significantly increased the *P_r_* value of both gelled emulsion and non-gelled emulsion (*P* < 0.05). Also, the *P_r_* value of gelled emulsion samples were significantly higher than those of non-gelled emulsion samples. In addition, entrapping emulsion in a gel matrix in combination with adding sesamol and sesamol esters to this system significantly enhanced the *P_r_* value (*P* < 0.05).

In gelled emulsion, the *P_r_* values of samples containing sesamol + sesamol ester were significantly lower than corresponding samples containing sesamol esters alone. In the non-gelled emulsion, the *P_r_* values of samples containing sesamol + SA and sesamol + SH were significantly lower than those samples containing SA and SH alone, while the *P_r_* value of sample containing sesamol + SB was slightly higher than that sample containing SB alone. Accordingly, sesamol esters did not show a synergistic effect with sesamol in gelled emulsion in the initiation phase. In the non-gelled emulsion samples, SA and SH did not show a synergistic effect with sesamol, while SB showed a slight synergistic effect with sesamol in the initiation phase of peroxidation.

### 3.4 Comparing antioxidant capacity of sesamol esters in gelled and non-gelled emulsions

The *AA* value of SA and SH in non-gelled emulsion samples were significantly higher than those of gelled emulsion samples (Table 3). In gelled emulsion, viscosity of continuous phase is higher than non-gelled emulsion. Viscosity can impact the transfer rate of antioxidant compounds to the active site of oxidation (Sato et al., 2014). Accordingly, the reduction in the efficiency of SA and SB in inhibiting peroxidation can be attributed to the role of mass transport phenomena on the antioxidant capacity of these antioxidants. In O/W emulsion, water soluble antioxidants can transfer among oil droplets by diffusing through the water phase, while water insoluble antioxidants can transfer among oil droplets by collision-exchange-separation transfer and micelle-assisted transfer pathways (Laguerre et al., 2017). The log *P* values of SA and SH were 1.38 and 3.62, respectively. Positive value of log *P* indicates that the molecule is more soluble in fat-like solvents, while negative value of log *P* indicates that the molecule is more soluble in water (Geetha, Singh, Deol, & Kaur, 2015). Thus, SA and SH are assumed to be transferred among oil droplets through collision-exchange-separation transfer and micelle-assisted transfer pathways. The high viscosity of continuous phase can limit the transfer of antioxidants to the oxidation site (Sun et al., 2007). Thus, the lower efficiency of sesamol esters in gelled emulsion during the propagation phase can be due to a decrease in their ability to move to the oxidation site by micelle-assisted transfer and collision-exchange-separation transfer pathways. The *AA* value of SB in gelled emulsion was higher than that of non-gelled emulsion. This may be due to the fact that SB with medium hydrophobicity (log P value of 2.61) is mainly located at the vicinity of interfacial area. Therefore, the mass transport phenomena had no significant effect on the efficiency of SB.

**Fig. 2:**
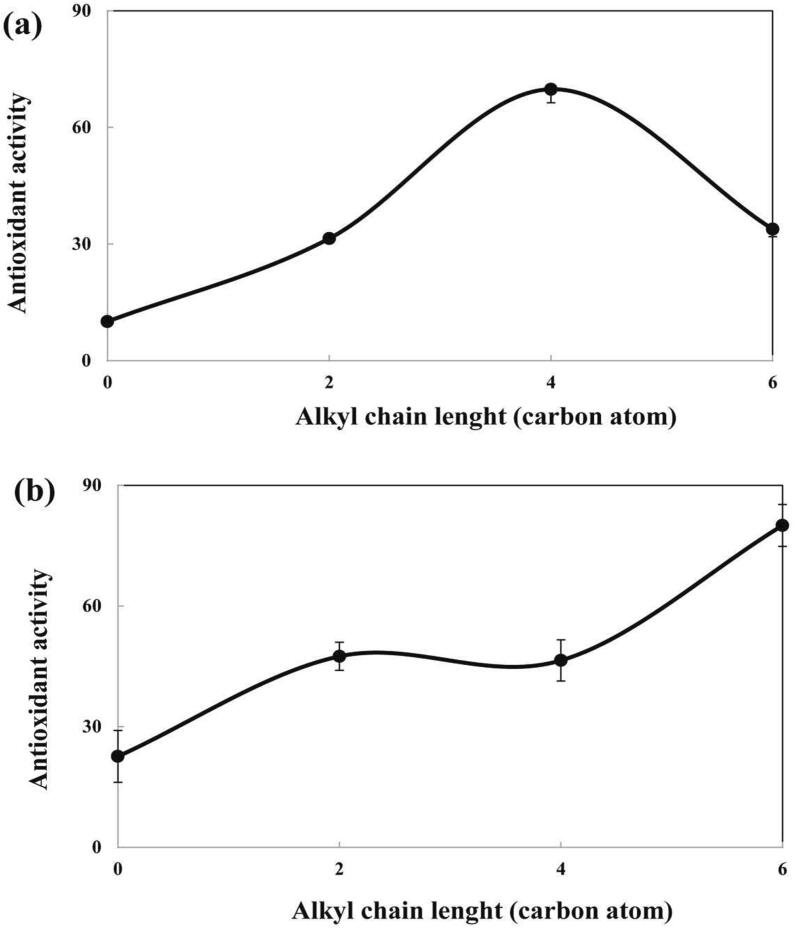
Relationship between alkyl chain length and antioxidant activity (AA) of sesamol esters in (a) gelled emulsion and (b) non-gelled emulsion.

Relationship between alkyl chain length of sesamol esters and *AA* value of sesamol esters is presented in Fig. 2. In gelled emulsion, by enhancing the chain length from 0 to 4 carbon atom, the *AA* value was enhanced. Further increase in alkyl chain length from 4 to 6 carbon atom decreased the *AA* value of sesamol ester. Accordingly, the relationship between *AA* value and alkyl chain length of sesamol esters in gelled emulsion was in accordance with the cut-off effect hypothesis. According to this hypothesis, the antioxidant activity enhances progressively by increasing chain length up to a critical point. At higher chain lengths, the activity of the antioxidants reduces. The reason for the cut-off effect theory for antioxidant molecules is that below a given hydrophobicity threshold, the antioxidant molecules are not close enough to the interfacial area. The reduction in antioxidant activity beyond a certain hydrophobicity can be explained by three mechanisms of “reduced mobility”, “internalization”, and the “self-aggregation” hypotheses. According to the “reduced mobility” hypothesis, the mobility of the antioxidant ester reduces in response to an increase in the alkyl chain length, thereby resulting in a lower tendency for moving toward the oil–water interface. Based on the internalization hypothesis, enhancing the alkyl chain length from the medium to long chains can result in driving the antioxidants away from the interfacial area into the core of the oil droplets. The “self-aggregation” hypothesis states that antioxidant esters with high hydrophobicity possibly exist as micelles in the water phase. Consequently, the concentration of antioxidant esters with high hydrophobicity at the interfacial area decreases (Laguerre et al., 2015). According to these explanations, the higher efficiency of SB than SA and SH can be related to the higher affinity of SB for locating at the interfacial area.

In non-gelled emulsion, the *AA* value of sesamol esters was increased as the chain length increased from 0 to 6 carbon atom. Therefore, the cut-off effect was disappeared in non-gelled emulsion. Panya et al. (2012) have stated that when surfactant micelles are absent in the water phase, the cut-off effect was observed and butyl rosmarinate and dodecyl rosmarinate showed higher antioxidant activity than eicosyl rosmarinate ester. When surfactant micelles were present, the efficiency of eicosyl rosmarinate enhanced and the cut-off effect was vanished. It was proposed that the surfactant micelles carried eicosyl rosmarinate from the core of the oil droplet to the oil–water interface, where they can inhibit peroxidation more efficiently (Panya et al., 2012). In this study, Tween 80 was used above its CMC value both in gelled emulsion and non-gelled emulsion. When surfactants are used above their CMC value, surfactant micelles form in the water phase. As a consequence, in non-gelled emulsion, surfactant micelles can transfer SH from the oil droplet core to the interfacial region and enhances the antioxidant capacity of SH. However, in non-gelled emulsion, the high viscosity of water phase can limit the ability of surfactant micelles to transfer SH to the interfacial area and the cut-off effect is still observed.

### 3.5 Oxidation kinetic parameters in the propagation phase

The *PP* value of the control gelled emulsion sample was 1.80-fold higher than that of control non-gelled emulsion sample (Table 4). Thus, entrapping oil droplets in a gel matrix enhanced the oxidative stability during the propagation phase as in the initiation phase. The *PP* value of SA, SB, and SH were higher than the control sample in gelled emulsion and non-gelled emulsion. Therefore, all sesamol ester molecules were not consumed during the initiation phase and parts of sesamol esters molecules remained active during the propagation phase. *T_p_* value is the time when the rate of ROOH formation reaches its highest value (*K_max_*). After this point, the decomposition reaction of ROOH begins. The *K_n_* value can use as a measure of oxidizability during the propagation phase (Farhoosh, 2020). According to the two-way ANOVA, *K_n_* values of gelled emulsion samples were significantly lower than those of non-gelled emulsion samples (*P* < 0.05). Also, adding sesamol, SA, SB, SH, and their combinations, significantly decreased the *K_n_* value. In addition, entrapping O/W emulsion in a gel matrix in combination with adding sesamol and sesamol esters to this system significantly decreased the *K_n_* value (*P* < 0.05).

**Table 4:** Oxidation kinetic parameters of the propagation stage of emulsion gel and non-gelled emulsion samples oxidation.

**Kinetic parameter**	**Control**	**S**	**SA**	**SB**	**SH**	**S + SA**	**S + SB**	**S + SH**
**Emulsion gel**								
***PP* (h)**	146.71 ± 2.12^c^	172.68 ± 21.16^bc^	275.73 ± 11.67^ab^	192.88 ± 14.75^abc^	208.00 ± 17.39^abc^	290.05 ± 23.43^a^	171.78 ± 4.80^bc^	274.28 ± 2.92^ab^
***T_p_* (h)**	92.86 ± 1.59^e^	183.95 ± 17.47^d^	291.61 ± 21.57^b^	389.23 ± 3.97^a^	382.24 ± 11.04^a^	220.53 ± 0.28^c^	268.77 ± 1.98^b^	210.18 ± 1.68^c^
***K_max_* × 10^2^(mM h^−1^)**	31.79 ± 1.31^b^	11.09 ± 1.52^d^	11.32 ± 2.02^d^	7.77 ± 3.06^d^	31.96 ± 1.42^b^	8.06 ± 0.43^d^	57.16 ± 1.77^a^	23.63 ± 0.53^c^
***K_n_* × 10^3^ (h^−1^)**	5.69 ± 0.08^a^	3.28 ± 0.00 ^cd^	2.90 ± 0.14 ^cd^	2.69 ± 0.08 ^cd^	3.96 ± 0.38^bc^	2.11 ± 0.06^d^	5.14 ± 0.14^ab^	2.87 ± 0.02 ^cd^
***W_f_* × 10^3^ (h^−1^)**	22.77 ± 0.32^a^	13.12 ± 0.00 ^cd^	11.58 ± 5.60 ^cd^	10.75 ± 0.29 ^cd^	15.83 ± 1.53^bc^	8.43 ± 0.22^d^	20.57 ± 0.57^ab^	11.48 ± 0.09 ^cd^
***W_d_* × 10^5^ (mM^-1^h^−1^)**	40.80 ± 0.55^a^	39.15 ± 5.39^a^	30.50 ± 7.48^ab^	39.92 ± 13.75^a^	19.62 ± 2.92^bc^	22.06 ± 0.00^bc^	18.50 ± 0.45^bc^	13.94 ± 0.10^c^
***ET_pp_* (h)**	180.69 ± 0.37	336.41 ± 17.47^c^	487.20 ± 73.06^b^	575.32 ± 1.07^a^	509.22 ± 23.31^b^	457.82 ± 6.59^b^	366.05 ± 4.66^c^	384.43 ± 0.34^c^
***E_p_***	–	1.18 ± 0.16^a^	1.89 ± 0.82^a^	1.32 ± 0.12^a^	1.42 ± 0.10^a^	1.98 ± 0.19^a^	1.17 ± 0.02^a^	1.87 ± 0.01^a^
***ORR_f_***	–	0.58 ± 0.01^bc^	0.51 ± 0.05^bc^	0.47 ± 0.01^bc^	0.69 ± 0.06^ab^	0.37 ± 0.01^c^	0.90 ± 0.01^a^	0.50 ± 0.00^bc^
***ORR_d_***	–	0.96 ± 0.12^b^	0.75 ± 0.17^ab^	0.98 ± 0.33^a^	0.48 ± 0.08^bc^	0.54 ± 0.01^bc^	0.45 ± 0.02^bc^	0.34 ± 0.00^c^
***IE_f_***	–	2.04 ± 0.31^ab^	4.67 ± 0.50^ab^	2.79 ± 0.22^ab^	2.05 ± 0.31^ab^	5.36 ± 0.73^a^	1.30 ± 0.04^b^	3.71 ± 0.04^ab^
***IE_d_***	–	1.25 ± 0.32^c^	2.73 ± 0.73^bc^	1.45 ± 0.60^c^	3.00 ± 0.70^bc^	3.66 ± 0.30^b^	2.58 ± 0.13^bc^	5.47 ± 0.01^a^

**Non-gelled emulsion**								
***PP* (h)**	81.40 ± 2.47^d^	86.96 ± 8.62^d^	227.33 ± 6.59^b^	267.40 ± 5.55^ab^	182.14 ± 14.17^c^	60.31 ± 0.59^d^	273.87 ± 3.28^a^	270.07 ± 46.84^ab^
***T_p_* (h)**	46.01 ± 1.00^c^	110.40 ± 3.73^b^	195.92 ± 10.93^a^	212.50 ± 2.70^a^	185.58 ± 9.98^a^	49.84 ± 4.21^c^	207.35 ± 4.61^a^	207.07 ± 25.96^a^
***K_max_* × 10^2^(mM h^−1^)**	73.09 ± 3.28^b^	37.64 ± 5.76^c^	21.02 ± 2.58^d^	20.55 ± 1.0^d^	21.94 ± 6.07^d^	107.57 ± 0.00^a^	15.97 ± 1.96^d^	18.30 ± 5.98^d^
***K_n_* × 10^3^ (h^−1^)**	10.65 ± 0.24^b^	7.70 ± 0.01^c^	3.40 ± 0.01^e^	3.01 ± 0.00^e^	4.24 ± 0.71^d^	14.16 ± 0.00^a^	2.89 ± 0.11^e^	3.13 ± 0.51^e^
***W_f_* × 10^3^ (h^−1^)**	42.58 ± 0.95^b^	30.81 ± 0.00^c^	13.62 ± 0.00^e^	12.03 ± 0.00^e^	16.96 ± 2.82^d^	56.66 ± 0.00^a^	11.55 ± 0.43^e^	12.53 ± 2.05^e^
***W_d_* × 10^5^ (mM^-1^h^−1^)**	62.02 ± 0.00^b^	63.79 ± 9.76^b^	22.22 ± 2.72^d^	17.62 ± 0.90^d^	33.00 ± 1.78^c^	74.61 ± 0.00^a^	20.95 ± 1.01^d^	21.76 ± 0.08^d^
***ET_pp_* (h)**	93.00 ± 0.06^e^	175.31 ± 3.73^d^	342.79 ± 10.93^b^	378.79 ± 2.67^a^	305.12 ± 9.91^c^	85.14 ± 4.21^e^	380.69 ± 1.86^a^	368.83 ± 0.52^a^
***E_p_***	–	1.07 ± 0.14^d^	2.79 ± 0.00^b^	3.29 ± 0.17^ab^	2.24 ± 0.24^c^	0.74 ± 0.03^d^	3.36 ± 0.06^a^	3.31 ± 0.47^ab^
***ORR_f_***	–	0.72 ± 0.02^b^	0.32 ± 0.01 ^cd^	0.28 ± 0.01^d^	0.40 ± 0.07^c^	1.33 ± 0.03^a^	0.27 ± 0.00^d^	0.30 ± 0.04^d^
***ORR_d_***	–	1.03 ± 0.16^b^	0.36 ± 0.04^d^	0.28 ± 0.01^d^	0.53 ± 0.03^c^	1.20 ± 0.00^a^	0.34 ± 0.02^d^	0.35 ± 0.00^d^
***IE_f_***	–	1.48 ± 0.22^d^	8.73 ± 0.21^bc^	11.65 ± 0.86^ab^	5.77 ± 1.69^c^	0.56 ± 0.03^d^	12.41 ± 0.04^a^	11.49 ± 3.24^ab^
***IE_d_***	–	1.06 ± 0.30^e^	7.86 ± 0.95^c^	11.57 ± 0.00^a^	4.23 ± 0.68^d^	0.62 ± 0.02^e^	9.98 ± 0.66^ab^	9.43 ± 1.32^bc^

^***^ Mean ± SD (*n* = 3). In each row, means with different superscript letters are significantly different (*P* < 0.05).

*S: sesamol, SA: sesamyl acetate, SB: sesamyl butyrate, SH: sesamyl hexanoate.

^**^ET_pp_: end time of the propagation stage, *IE_f_*: inhibitory activity against the ROOHs formation, *IE_d_*: inhibitory activity against the *ROOHs* decomposition, *W_f_*: pseudo-first order rate constant of *ROOHs* formation at the propagation stage, *E_p_*: antioxidant effectiveness during the propagation stage, *W_d_*: pseudo-second order rate constant of *ROOHs* decomposition at the propagation stage, *K_max_*: maximum rate of lipid hydroperoxide formation in the propagation stage, *ORR_f_*: oxidation rate ratio of *ROOHs* formation during the propagation stage, *ORR_d_*: oxidation rate ratio of *ROOHs* decomposition during the propagation stage, *PP*: duration of the propagation stage, *K_n_*: normalized form of maximum rate of lipid hydroperoxides formation in the propagation stage, *T_p_*: occurrence time of maximum rate of lipid hydroperoxide formation at the propagation stage.

SA, SB, and SH were more effective than sesamol in enhancing the *T_p_* value and reducing the *K_n_* value in gelled emulsion and non-gelled emulsion. This indicates the higher capacity of sesamol esters than sesamol in inhibiting peroxidation during the propagation phase. This result can be related to the better interfacial performance of sesamol esters than sesamol in gelled emulsion and non-gelled emulsion. Samples containing sesamol + SB showed higher *T_p_* value than those samples containing sesamol + SA and sesamol + SH in gelled emulsion and non-gelled emulsion.

The *E_p_* value shows the effectiveness of sesamol, sesamol esters, or sesamol + sesamol esters in inhibiting peroxidation during the propagation phase. The *ORR_f_* value is the ratio between *W_f_* value of sample containing antioxidant to the control sample and *ORR_d_* value is the ratio between *W_d_* value of the sample containing antioxidant to the control sample. The *IE_f_* value simultaneously evaluates *Ep* and *W_f_* values and the *IE_d_* value simultaneously evaluates *Ep* and *W_d_* values. The *IE_f_* value of gelled emulsion samples containing SA, SB, and SH were 2.29-, 1.13-, and 1.01-fold higher than gelled emulsion samples containing sesamol, respectively, while the *IE_f_* value of non-gelled emulsion samples containing SA, SB, and SH were 5.90-, 7.87-, and 3.90-fold higher than sample containing sesamol, respectively. Also, the *IE_d_* value of gelled emulsion samples containing SA, SB, and SH were 2.18-, 1.16-, and 2.40-fold higher than sample containing sesamol, respectively, while the *IE_d_* value of non-gelled emulsion samples containing SA, SB, and SH were 7.41-, 10.92-, and 3.99-fold higher than sample containing sesamol, respectively. Accordingly, sesamol esters exhibited higher efficiency in inhibiting the formation and decomposition of ROOHs in non-gelled emulsion than gelled-emulsion during the propagation phase. This reduction in the efficiency of sesamol esters in inhibiting peroxidation during the propagation phase can be attributed to the high viscosity of gelled emulsion which can limit the ability of surfactant micelles to transfer sesamol esters to the oil–water interface.

## 4 Conclusion

The goal of this study was to investigate the efficiency of sesamol, SA, SB, and SH in gelled emulsion in comparison with non-gelled emulsion to assess the role of mass transfer on their antioxidant capacity in the initiation and propagation phase of peroxidation. Esterification of sesamol increased the *AA* value of sesamol in gelled emulsion and non-gelled emulsion, which indicates the higher interfacial performance of sesamol esters than sesamol in gelled emulsion and non-gelled emulsion. In addition, the *AA* values of SA and SH in gelled emulsions were significantly lower than those of non-gelled emulsion. This can be due to the limited ability of SA and SH to move toward the oil–water interface, where oxidation occurs. In contrast, SB exhibited higher *AA* value in gelled emulsion than non-gelled emulsion. This indicates that entrapping oil droplets in a gel matrix do not have any negative effect on the efficiency of antioxidants with medium hydrophobicity, which are mainly concentrated at the vicinity of interfacial area. In gelled emulsion the cut-off effect was observed and a nonlinear relationship was observed between antioxidant activity of sesamol esters and their alkyl chain length. In non-gelled emulsion, the cut-off effect was vanished and antioxidant activity of sesamol esters increased by enhancing the alkyl chain length. In general, the antioxidant activity of antioxidants that are expected to locate far from the interfacial area in gelled emulsions was lower than non-gelled emulsion, while the antioxidant activity of antioxidants that are expected to locate at the vicinity of the interfacial area in gelled emulsions was higher than non-gelled emulsion.

## CRediT authorship contribution statement

**Malihe Keramat:** Conceptualization, Investigation, Data curation, Methodology, Formal analysis, Software, Resources, Validation, Visualization, Writing – original draft. **Mohammad-Taghi Golmakani:** Funding acquisition, Project administration, Supervision, Writing – review & editing. **Mehrdad Niakousari:** Funding acquisition, Project administration, Supervision, Writing – review & editing. **Mohamad Reza Toorani:** Conceptualization, Methodology, Formal analysis, Software, Resources.

## Declaration of Competing Interest

The authors declare that they have no known competing financial interests or personal relationships that could have appeared to influence the work reported in this paper.

## Data Availability

Data will be made available on request.
