# Supplementary figures and images for: Comparison of the antioxidant capacity of sesamol esters in gelled emulsion and non-gelled emulsion
Source: Food Chem X. 2023 May 3;18:100700. doi: 10.1016/j.fochx.2023.100700 (PMC10189410; doi:10.1016/j.fochx.2023.100700)

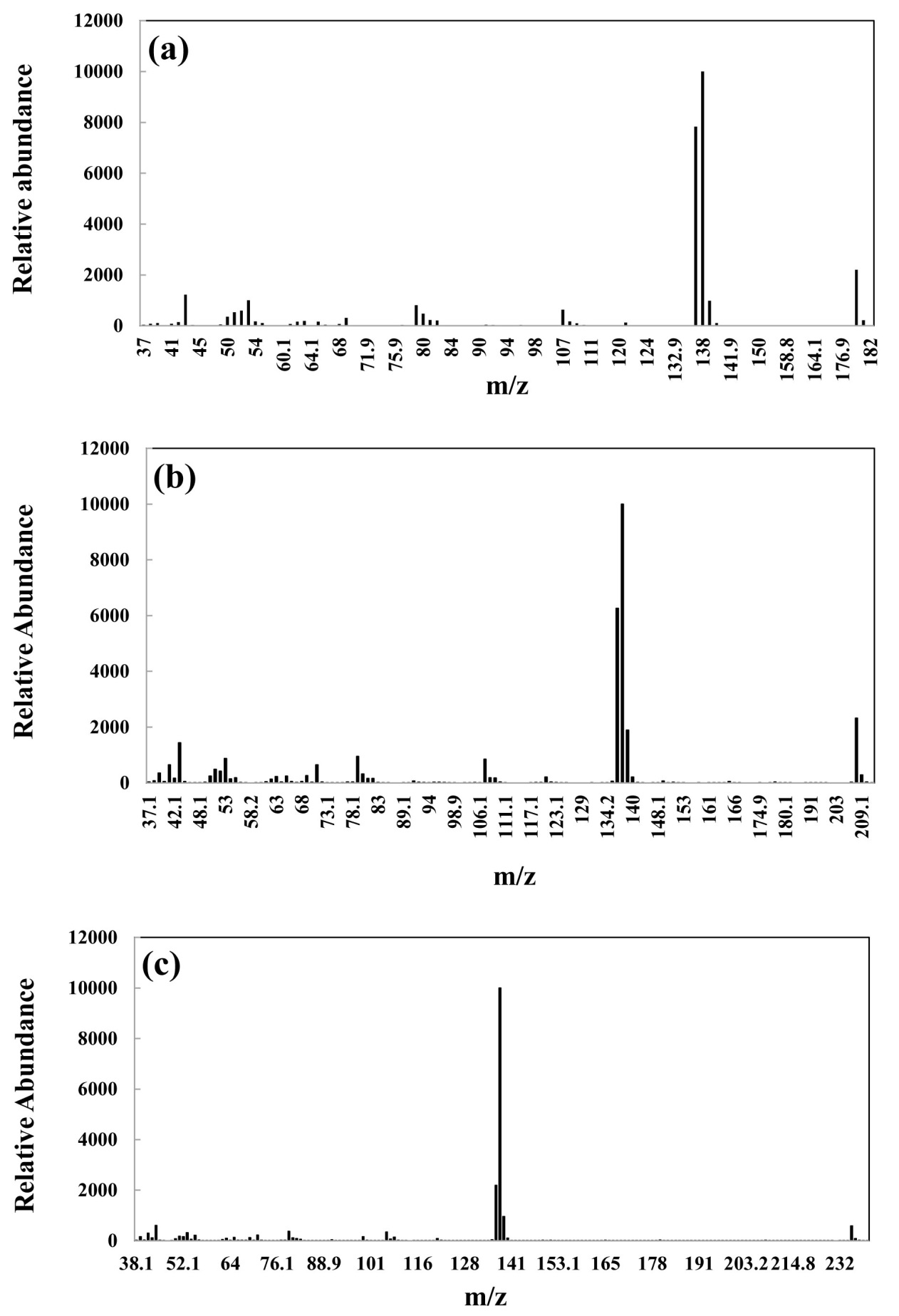


**Fig. 1S.** Mass spectra of (a) sesamyl acetate, (b) sesamyl butyrate, and (c) sesamyl hexanoate.

Supplement: Supplementary data 1 [file mmc1.docx]
